# An Expert Panel Review of Endoscopic Vein Harvesting Devices: Benefits, Limitations, and Clinical Insights

**DOI:** 10.1093/icvts/ivaf204

**Published:** 2025-09-02

**Authors:** Bhuvaneswari Krishnamoorthy, Sam Raaj, Andjela Susanj, Gianluca Adinolfi, Donna Croft, Asher G Joseph, Michael L Sullivan, Thuy Le, Matthew Petrides, Chris Darst, Richard M Vitali, Igor Zivkovic, James B Barnard

**Affiliations:** Directorate of Nursing and Midwifery, The University of Salford, Manchester, M6 6PU, United Kingdom; Manchester University NHS Foundation Trust, Wythenshawe Hospital, Manchester, M23 9LT, United Kingdom; Department of Cardiovascular Sciences, Faculty of Health, Biology and Medicine, University of Manchester, Manchester, M13 9PL, United Kingdom; Faculty of Medicine, Medical School, Imperial Medical College, London, SW7 2AZ, United Kingdom; Department of Cardiovascular Institute Dedinje, Belgrade, 11040, Serbia; Faculty of Medicine, University of Belgrade, Belgrade, 11000, Serbia; Department of Cardiothoracic Surgery, Guy’s and St Thomas NHS foundation Trust, Royal Brompton Hospital, London, SW3 6NP, United Kingdom; Department of Cardiothoracic Surgery, Blackpool Teaching Hospitals NHS Foundation Trust, Blackpool, FY3 8NR, United Kingdom; Manchester University NHS Foundation Trust, Wythenshawe Hospital, Manchester, M23 9LT, United Kingdom; Department of Cardiothoracic Surgery, Sarasota Memorial Healthcare System, Sarasota, FL, 34239, United States; Department of Cardiothoracic Surgery, University of Michigan Medical Center, Michigan, MI, 48109, United States; Department of Cardiac Surgery, Jersey Shore University Medical Center, NJ, 07754, United States; Department of Cardiothoracic Surgery, CHI Health Nebraska Heart Hospital, Lincoln, NE, 68046, United States; Department of Cardiac Surgery, Maimonides Medical Center, State University of New York, NY, 11219, United States; Department of Cardiovascular Institute Dedinje, Belgrade, 11040, Serbia; Faculty of Medicine, University of Belgrade, Belgrade, 11000, Serbia; Manchester University NHS Foundation Trust, Wythenshawe Hospital, Manchester, M23 9LT, United Kingdom

**Keywords:** endoscopic vessel harvesting, coronary artery bypass grafting, device, harvesters

## Abstract

**Objectives:**

Endoscopic vessel harvesting (EVH) devices are technically complex and the learning curve for novice practitioners can be steep, due to the need for refined hand-eye coordination and device familiarity. Training and mentoring approaches vary widely, as does the experience level of practitioners entering EVH practice.

**Methods:**

This expert review was conducted by 10 international EVH specialists from the United Kingdom, United States, and Serbia, each with 18 to 28 years of experience. Comprehensive searches of EMBASE, Cochrane, PubMed, CINAHL, and Google Scholar revealed no head-to-head comparative studies of EVH devices. As a result, the group evaluated EVH device industry specifications and white papers to analyse the evolution, component features, and limitations of current systems. Expert consensus was also sought to outline ideal device attributes and training enhancements.

**Results:**

Studies suggest the EVH learning curve ranges from 5 to 30 cases; however, studies have reported that even after 100 cases, learning may be incomplete, particularly when assessed using optical coherence tomography for conduit injury. A lack of high-quality comparative studies and wide variability in device design, institutional practices, and user experience hinder conclusions about the superiority of any specific EVH system. Device choice is often based more on training background and availability than on clinical evidence.

**Conclusions:**

No existing studies link specific device-related learning curves to clinical outcomes or conduit quality. There is an apparent need for independent device evaluation, standardized training programmes, and robust comparative outcome data to support evidence-based device selection that prioritizes patient safety and conduit long-term patency.

## Graphical abstract

**Figure:**
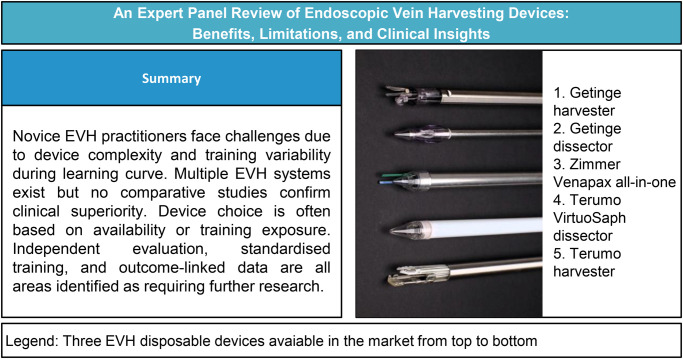

## INTRODUCTION

Coronary artery bypass graft (CABG) surgery is the gold standard treatment choice for multiple vessel or left main coronary artery disease (CAD).^1^^,^^2^ Arterial grafts offer superior patency over venous conduits, owing to their favourable biological and haemodynamic properties.^3^ Contemporary evidence consistently supports multivessel arterial grafting as being associated with improved surgical outcomes.^4^^,^^5^ Most used arterial conduits are the internal mammary artery (IMA) and radial artery (RA), while venous grafts typically involve the long and short saphenous veins (LSV and SSV). Less frequently, the gastroepiploic, superior and inferior epigastric, and splenic arteries are employed.^3^

## CONDUITS IN CORONARY SURGERY

IMA was first recognized in 1946, with its clinical utility documented in the literature by 1986.^6^ It is now considered the gold standard for CABG due to its durability and superior patency, reported at 93%-96% at 1 year, 88%-98% at 5 years, and 85%-95% beyond 10 years.^5^ RA was initially introduced by Carpentier in 1971, was temporarily abandoned due to its propensity for vasospasm and early occlusion.^7^ However, it was reintroduced in 1992 after better understanding of its physiological behaviour and protective strategies. Recent advancements, including endoscopic harvesting and “no-touch” technique combined with pharmacological vasodilators, have improved its performance, with reported patency rates of 89%-92% at 1 year, 90%-98% at 5 years, and 89%-91% beyond 10 years.^5^^,^^8^ The LSV, introduced by René Favaloro in 1968, remains a widely used graft owing to its sufficient length and accessibility for multivessel revascularization.^3^ However, its patency is comparatively lower, ranging from 81% to 97.9% at 1 year, 75% to 86% at 5 years, and declining to 50% to 60% beyond 10 years.^5^

LSV is a well-established choice of conduit for multivessel CAD not affecting the left anterior descending coronary artery. Endoscopic vein harvesting (EVH) is widely used due to its reduced postoperative morbidity and better patient satisfaction.^9^^,^^10^ EVH has evolved significantly over the years, particularly in the context of CABG surgery. The transition from open harvesting technique’s to EVH was adapted to reduce hospital stay, infection rates, postoperative pain, and better cosmetic scarring.^10^^,^^11^ This review has been compiled by 10 international experts in EVH, each with a minimum of 18-28 years of experience and having performed between 2500 and 6000 endoscopic procedures.

## NON-TOUCH PEDICLE AND SKELETONIZED APPROACH

To prevent or delay saphenous vein graft thrombosis and atherosclerotic changes leading to graft failure,^12^ optimal graft preparation is essential. Intraoperative injury can disrupt the intima and trigger biochemical and functional changes that impair long-term viability.^13^ Techniques such as skeletonization, mechanical manipulation, and adventitial stripping, whether via endoscopic or open technique, can damage the endothelium and promote inflammation, contributing to graft occlusion.^14^ The no-touch technique involves harvesting the saphenous vein as a pedicled graft, preserving the vein wall’s structural and functional integrity, including the vasa vasorum, perivascular nerves, and surrounding adipose tissue.^15^

The 2018 European Society of Cardiology/European Association for Cardiothoracic Surgery myocardial revascularization guidelines support this technique with a Class IIa recommendation.^16^ Several studies suggest it offers patency rates comparable to arterial grafts^15–19^; however, recent Randomised Control Trials (RCT) and cohort analyses have shown no clear advantage in patency or clinical outcomes.^19–21^ Proper graft handling during CABG promoting no-touch techniques, avoiding mechanical distension, and ensuring meticulous anastomosis is critical to minimizing graft degeneration.^3^ Nevertheless, structured education on graft preservation and intraoperative handling is still lacking in some EVH training curriculum, underscoring the need to integrate these concepts into formal surgical education.

## EVOLUTION OF ENDOSCOPIC CONDUIT HARVESTING

EVH requires the use of specialized endoscopic equipment and proficiency in specific minimally invasive techniques. Successful EVH is dependent on adequate training to ensure the consistent procurement of high-quality conduits.^22^^,^^23^ In 2022, the global EVH market was valued at $507.3 million, and it is estimated to grow at a compound annual growth rate of 3.5% from 2023 to 2031 and reach $686.6 million by 2031.^24^ A variety of surgical companies have developed single-use or reusable endoscopic device systems. These can be broadly categorized as follows: major multinational single-use EVH system manufacturers such as Getinge (Maquet, Sweden), Terumo Corporation (Japan), Zimmer Venapax Medical Inc (USA), Johnson & Johnson (USA), Sorin International, and LivaNova Plc (UK); device-specific reusable product developers including Karl Storz Medical (Bisleri Model, Tuttlingen, Germany).^24^

The first clinical use of an EVH system was developed by Maquet in 1996, and this company is still the major players in the market with over 2 500 000 EVH procedures completed in 2018.^25^ While there are now multiple systems in the market, the evidence for the superiority of one system over another clinically remains uncertain.

## TIMELINE OF EVH

In the 1990s: Prior to the advent of dedicated endoscopic instruments in 1995, various modified tools were employed for LSV harvesting, including the modified Mayo vein stripper,^26^ mediastinoscopy instruments,^27^ the Mini-Harvest system which is a basic device comprising a retractor with a light source^28^ (Auto suture, USA surgical corporation, Norwalk, CT), laparoscopic equipment^29^ (Snowden and Pencer, Tucker, GA, USA), and the Ethicon Endopath system^30^ (Endo-surgery, Inc, Cincinnati, OH). However, many of these devices were subsequently withdrawn from clinical use due to limitations such as inadequate visualization, cumbersome instrumentation, poor ergonomics, increased perioperative complications, saphenous neuralgia and, most notably, suboptimal conduit quality which required numerous repairs.^26–30^

2000 onwards: Several major surgical device manufacturers have progressively refined EVH systems, transitioning from multi-component setups to single-handed, integrated devices that enhance visualization, minimize tissue trauma, and improve procedural efficiency.^25^^,^^31^ By 2017, EVH was established as the standard practice in the United States, following a consensus statement by the International Society of Minimally Invasive Cardiac Surgery.^32^ However, adoption outside of the U.S. remained limited due to quality of the conduit and added cost of the device.

## CRITICAL EVIDENCE IN 2009

A pivotal 2009 study in the New England Journal of Medicine analysed 3000 CABG patients from the PREVENT-IV trial and reported higher 1-year angiographic graft failure and increased 3-year mortality in patients who underwent EVH (*n* = 1753) compared to Open Vein Harvesting (OVH) (*n* = 1247).^33^ All grafts in PREVENT-IV underwent *ex vivo* pressurized drug or placebo delivery, contributing to overall higher graft failure rates than in other CABG trials. Nonetheless, a post-hoc propensity-adjusted analysis found EVH was associated with a significantly increased risk of death (HR 1.5; 95% CI, 1.1-2.0; *P* < 0.005) and of the composite end-point of death, MI, or repeat revascularization (HR 1.22; *P* = 0.04).^34^

In response, the United Kingdom’s National Institute for Health and Care Excellence (NICE) recommended that EVH be performed only under special clinical governance, with informed consent and audit or research oversight.^35^ These concerns prompted suspension of EVH programmes in several UK and European centres. Adoption rates at the time varied significantly, with over 80% of North American patients receiving EVH versus under 50% in Europe.^36^ The findings sparked debate over key variables including device type, operator skill, patient selection, and comorbidities.^37^^,^^38^ Subsequent studies and meta-analyses have supported EVH’s benefits, including fewer wound complications, higher patient satisfaction, and shorter hospital stays.^39^^,^^40^

## BETTER EVH SYSTEM vs CONDUIT QUALITY

There are different types of endoscopic devices on the market, and it is hard to choose which provides a better conduit quality. Harvesters may only receive EVH information from surgical company representatives, which could lead to biased perspectives favouring their own device. Additionally, the use of diverse EVH devices and techniques increases the complexity of the procedure, influencing and impacting procedural efficacy. Maintaining the integrity of conduits is a significant concern for practitioners involved in harvesting to achieve better outcomes.^9^ Reassuringly, a Randomized Endo-Vein Graft Perspective (REGROUP) study with a median follow-up of 4.7 years involving 1150 participants (576 randomized to EVH and 574 to OVH) found no significant difference in the incidence of major adverse cardiac events (MACE) between the 2 groups. MACE occurred in 21.9% of the EVH group and 23.5% of the OVH group (HR 0.92; 95% CI, 0.72-1.18; *P* = 0.52).^39^ Still concerns persist regarding graft quality, endothelial integrity, and long-term outcomes.^38–40^ These concerns were supported by a survey conducted in the United Kingdom by Soni et al,^36^ which concluded that conduit quality and cost considerations were the primary concerns for surgeons adopting EVH into clinical practice. EVH is considered as more cost effective than OVH with the savings of $92.33 and quality adjusted life-year gains of 0.206 per patient over a lifetime.^41^ A study that followed up Veterans Affairs patients stated that mean costs plateaued at approximately $20 000 through year 7 with a slight increase in mean costs between 7 and 10 years.^42^ An important point raised by Ryan and Iribarne^43^ must be taken into consideration that whether these negated cost savings from reduced wound complications can compensate for the increased follow-up care costs due to adverse cardiac event rates with EVH.^36–41^

Our EVH experts emphasize that the early acquisition of hand-eye coordination skills, proficiency in the handling and precise manipulation of advanced endoscopic equipment, and a thorough understanding of the implications of vein damage are critical components in structured training. When combined with formal certification, these elements are essential for ensuring the procurement of high-quality conduits in CABG surgery. This review aims to critically analyse the various types of endoscopic equipment available in clinical settings and offer a clear overview from international experts for harvesters to determine the ideal choice for their needs. Additionally, it will explore whether surgical companies need to make changes to the current endoscopic systems.

## OVERVIEW OF THE DISPOSABLE EVH SYSTEMS

A detailed description of each system evolution currently in use is illustrated in **Table S1** and the benefits and drawbacks of each system including expert tips are illustrated in **Table S2**. Some EVH devices are now historical after being withdrawn from the market. These include the Johnson & Johnson device, the Sorin Clear glide, and the LivaNova endoscopic vessel harvester system.

## TERUMO: OPEN TUNNEL CO_2_ SYSTEM

The Virtuo-Saph Plus system comprises a dissector, harvester, and optional trocar, distinguished by its open-tunnel design that avoids continuous pressurized CO_2_ insufflation. This approach mitigates risks associated with high-pressure insufflation such as intraluminal thrombus formation, vessel injury, CO_2_ embolism^44^, and hypercarbia^22^^,^^26^^,^^45^ by reducing mechanical stress on the vessel wall and limiting intraluminal pressure.

The dissector, measuring 40 cm in length, features an atraumatic conical tip encased in a polytetrafluoroethylene (PTFE) sheath. PTFE offers excellent biocompatibility and low friction, minimizing tissue trauma and microbial adherence critical in confined anatomical spaces of the lower limb.^46–48^ The endoscope tip includes orientation rings that assist with spatial navigation and distance estimation to the target vessel.^49^ CO_2_ is delivered directly through the endoscope tip to enhance visibility without tunnel pressurization. A trocar may be attached externally to the tunnel entrance for added stability without compromising vessel wall integrity.^49^

Expert users advocate a posterior-first dissection technique, allowing the vein to “settle” gravitationally within the tunnel before performing the anterior pass (**Video 1**: Terumo VirtuoSaph 00:04:14 to 00:06:33 seconds). In this system, the VasoView C-ring and 5-mm cautery were replaced by Terumo’s V-keeper, incorporating a V-lock and V-cut mechanism. This configuration maintains a consistent 9 mm distance between the stabilizer and cautery element, reducing the risk of thermal injury and subsequent intimal hyperplasia.^50^

The V-keeper tip stabilizes the vein via a smooth convex base that glides beneath the vessel, while its lateral arms secure the conduit through the V-lock mechanism. Two lateral notches isolate side branches, enhancing visibility and access. The integrated V-cutter allows simultaneous coagulation and branch transection, with the V-cautery switch enabling controlled energy delivery to minimize endothelial injury.

Additionally, the integrated endoscope wiper enables rapid removal of fat and blood, maintaining a clear visual field during dissection and branch division^49^ (**Video 2**).

## GETINGE: CLOSED TUNNEL CO_2_ SYSTEM

For the VasoView Hemopro 2 system, a skin incision of <2 cm is optimal to ensure a snug fit for the blunt-tip trocar (BTT) without inflation. If a larger incision is required, particularly during the learning phase, a purse-string suture is recommended to maintain seal integrity and preserve tunnel visualization. Inflation of the BTT should be avoided, as it may exert pressure on the proximal vein and impede blood flow during harvesting.^51^^,^^52^

The primary role of CO_2_ insufflation is to maintain tunnel patency and define tissue planes for dissection. While manufacturer guidelines recommend 3-5 L/min at 10-12 mmHg, our experts suggest a reduced flow of 1-3 L/min to prevent desiccation of the adventitial layer. A rate of 1 l/min is suitable for patients with thin legs, while 2-3 L/min may be more appropriate for those with higher body mass index (BMI).^53^

Heparin administration (2500-5000 units) prior to harvest reduces the risk of intraluminal thrombosis.^9^^,^^54^ Although some surgeons avoid heparin during IMA harvesting due to bleeding risk, we recommend case-by-case assessment, particularly for novice practitioners. A locally developed, multidisciplinary heparin bolus algorithm (**Figure S1**), in use since 2007 across multiple UK centres, can guide decision-making during early training.

EVH dissection generally follows 3 sequential steps: anterior, posterior, and lateral dissection of the vein and its branches (**Video 1**: Getinge Hemopro 00:00:01 to 00:02:06 seconds). In patients with elevated BMI, subcutaneous fat may act as a protective cushion, reducing the need for aggressive dissection and minimizing vein trauma. In contrast, low BMI patients with minimal adipose tissue are at higher risk of vein bruising or small-branch avulsion. In such cases, expert harvesters often adopt a “peeling-off” technique, dissecting the vein with its surrounding adventitia and perivascular sheath to preserve structural integrity.

For patients with small leg circumference (<17-19 cm), experienced practitioners often favour a posterior-lateral-anterior dissection sequence. This approach allows posterior and lateral space creation, enabling the vein to “fall away” from the equipment, reducing mechanical stress. Anterior or lateral fasciotomy should be performed before dissection to create tunnel space and prevent friction between the device and vein. During dissection in thin patients, the cone tip should remain angled upward and away from the vein to avoid direct trauma.^53^

Novice users must also be aware that improper handling or extended use of the Hemopro device, such as over-bending, twisting, or neglecting pre-use inspection, can lead to overheating or malfunction. Excess heat may damage the internal filament and impair cauterization. Our experts recommend using the Hemopro primarily for small-branch cautery during dissection, reserving larger or thicker branches for ligation at the end of the harvest to minimize device stress and reduce intra-tunnel bleeding.

For larger branches, the intermittent coagulation technique is preferred. This involves applying energy in three 5-second bursts, each separated by a 2-second pause, to ensure complete coagulation before dividing the vessel. The jaws should only be opened after full separation to avoid thermal damage and bleeding.^53^

To optimize safe and effective use, we propose a C.L.A.M.P. technique^51^:

- Clean the jaws: Regular cleaning enhances tissue sealing and minimizes smoke production; removal of excess adipose tissue also improves visualization.
- Locate the vessel: Align the jaws carefully towards the target branch, ensuring the active cautery surface faces away from the main vein to prevent collateral thermal injury.
- Activate energy: Only activate cautery when the vessel and branch are fully visible to reduce the risk of damage to adjacent structures.
- Mild tension: Apply gentle traction during coagulation to promote safe branch separation. Excessive pulling or twisting may cause avulsion or damage to the device.
- Proper fascia handling: During fascial dissection, higher tension may be applied safely, as fascia can withstand more force without increasing complication risk.

## ZIMMER BIOMET VENAPAX SYSTEM

The introduction of the Venapax dual-pass EVH system in 2013 marked a significant evolution in EVH instrumentation design. Unlike earlier platforms requiring multiple instruments or frequent switching between dissection and cautery tools, Venapax integrates blunt dissection, branch ligation, and spot cautery into a single, unitary device. In expert hands, this design streamlines workflow, reduces operative time, and minimizes conduit manipulation, particularly due to the elimination of a retraction arm.

Atraumatic dissection (**Video 1**: Zimmer Venapax 00:02:07 to 00:04:13 seconds) begins with posterior tunnelling at the 6 o’clock position beneath the vein, enabling gravitational clearance of minor bleeding and natural suspension of the anterior vein wall. This facilitates gentle anterior dissection. Following posterior tunnelling and tributary ligation, the device is withdrawn from the distal tunnel towards the trocar to identify and cauterize any bleeding points. At the trocar level, the conical tip advances along the 10 and 2 o’clock positions, maintaining dissection above the adventitial plane to preserve the vasa vasorum. Avoiding the 12 o’clock position further protects the anterior adventitia and associated microvasculature. This dual-pass technique allows the operator to ligate branches sequentially without advancing beyond them, reducing the risk of avulsion.

The system’s core features include a transparent fixed conical tip for blunt dissection and retractable rotational bipolar electrodes for precise cautery. Emphasizing a “minimal touch” approach, it limits direct vein handling and the number of device passes, thereby supporting endothelial preservation. The bipolar cautery mechanism delivers targeted thermal energy with minimal lateral spread; however, its effectiveness remains technique-dependent and requires real-time tissue response awareness. The learning curve varies according to individual manual dexterity.

## LEARNING CURVE AND ITS IMPACT

The learning curve period of each EVH device varies depending on the harvester’s surgical experience and manual dexterity.^9^ No study has been conducted to compare the differences in the learning curves between specific devices. Similarly, there is no study that we are aware of that has related the specific learning curve with each device to clinical outcomes or a measure of histologically apparent injury to the conduit with the use of one device compared to another. However, there are a few studies that have identified that the learning curve period ranges from 5 to 30 cases.^54^^,^^55^ In contrast, Kay et al^56^ and Desai et al^23^ found that learning was not complete with even close to 100 cases of experience when using conduit injury on optical coherence tomography (OCT) imaging as a more sensitive metric for learning. First, patient selection during learning plays a key role in enhancing the safety of EVH surgery.^53^^,^^54^ Second, surgical skills acquisition for endoscopic conduit harvesting requires prior detailed knowledge of the relevant anatomy, understanding the principles of conduit integrity and intimal preservation, training in proper usage of the equipment and understanding the potential complications encountered during the procedure.

Careful patient selection is essential during the initial EVH learning curve. Novice harvesters should avoid obese patients, as increased subcutaneous adiposity can obscure anatomical landmarks, complicating vein identification and increasing the risk of deviating from the correct dissection plane. This may lead to tissue disruption and procedural difficulty. Conversely, patients with thin legs, where the LSV is visible on examination and lies <3 mm beneath the skin on ultrasound, are also unsuitable for early training due to the risk of inadvertent injury. Similarly, patients with severe varicosities should be avoided, as excessive bleeding and poor tunnel visibility can compromise safe conduit harvesting.^53^^,^^57^

Oversight and engagement from experienced harvesters and how to tackle or ask for help when required is vital.^9^

## EXPERT TIPS FOR QUALITY CONDUIT HARVESTING DURING LEARNING CURVE PERIOD

**Figure 1.**
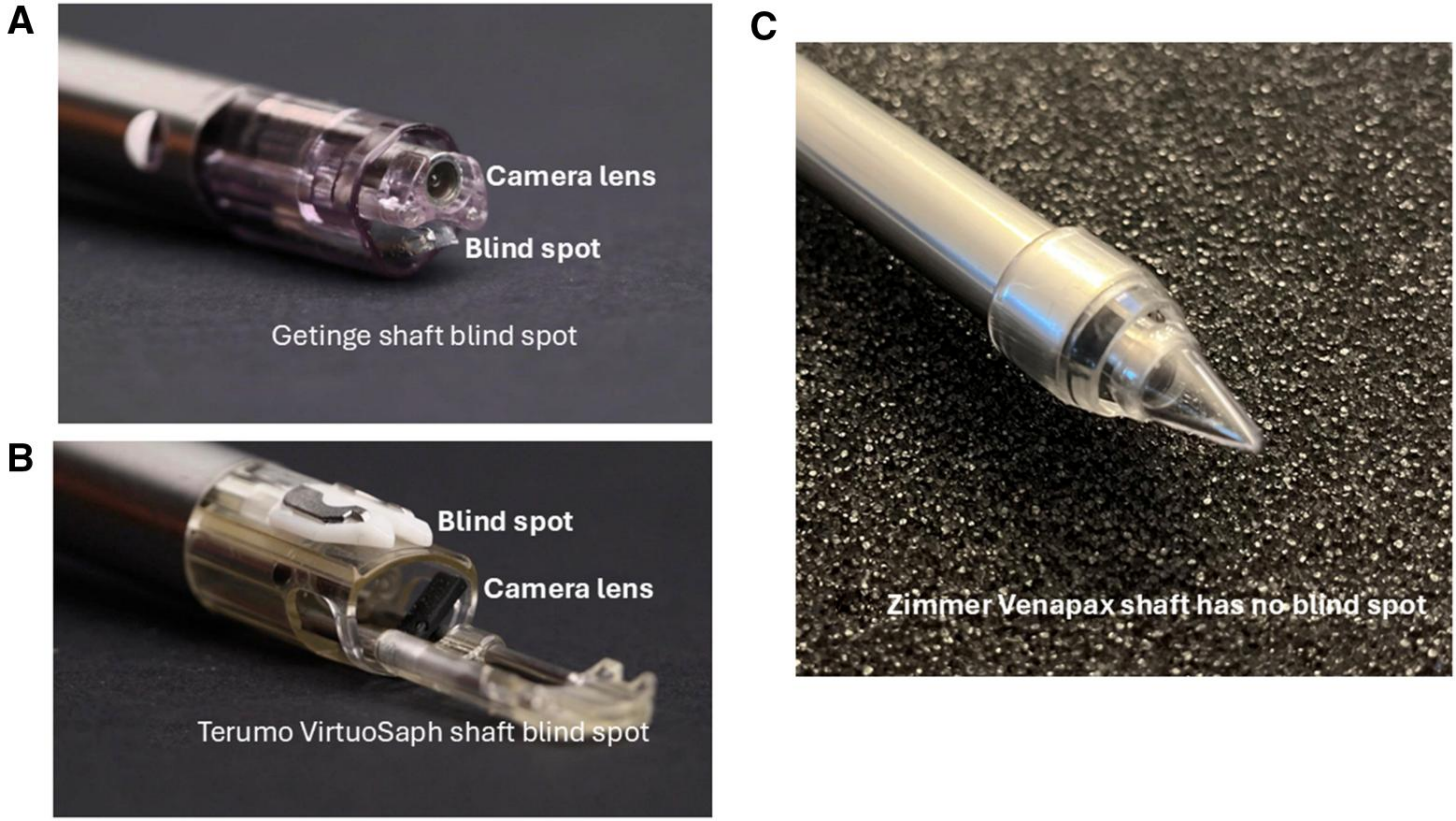
(A-C) Blind Spot on the EVH Device

**Figure 2.**
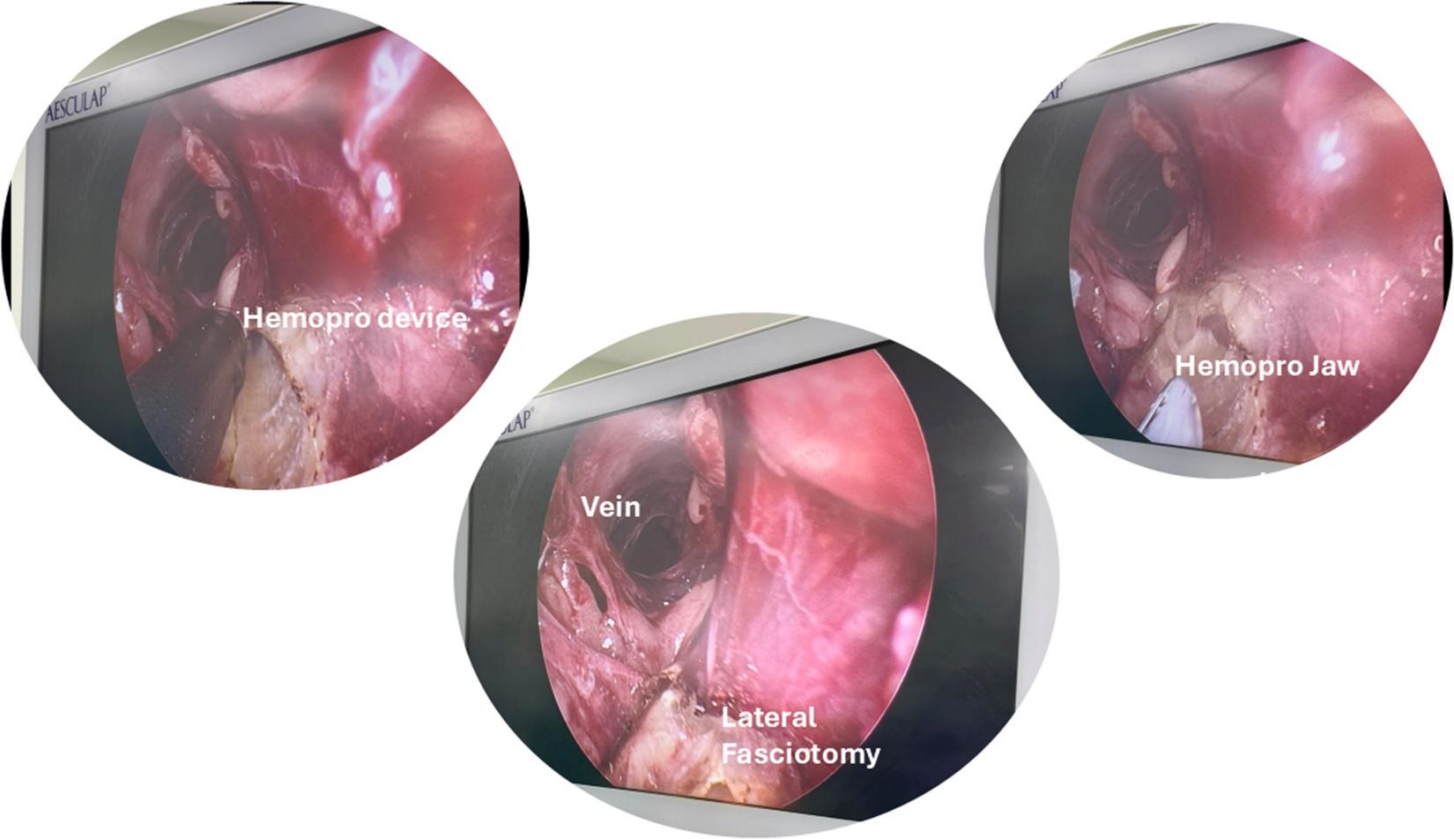
Fasciotomy to Create Space in the Tunnel Using Getinge Hemopro Device

1. Use of ultrasound: The use of ultrasound technology for vessel harvesting assessment prior to vein harvesting can help the EVH harvesters to understand the depth of the vein, location of large branches, the course of the vessel, any bifurcations/abnormalities, size of the vein, and where to make the skin incision. The LSV anatomical variations can range from 20% to 40%, which can be easily detected by the ultrasound guided scan, and this helps the harvesters to plan the surgical techniques and choose the appropriate limbs for harvesting.^52^^,^^58^ To check for thrombus on the LSV, a compression technique with the ultrasound probe is easily performed to confirm patency and exclude any thrombus. The LSV should be fully compressed under applied pressure, if there is a thrombus presence it will be incompressible. The vein should be compressed at regular intervals around every 5 cm along its length. If there is any thrombus, it will appear on the ultrasound in the lumen of the vein to be more echogenic/grey than blood. Fresh thrombus will appear black and chronic thrombus will appear white inside the vein.^59^^,^^60^
2. “Tip search” of the LSV on obese patients: It is recommended to enter the skin incision port at an angle between 90° and 45°, followed by a systematic search for the vessel in an arc-shaped pattern beginning anteriorly, then moving laterally, and finally posteriorly to minimize the risk of inadvertent vessel injury. The eye of the dissecting cone tip should be used in a manner similar to a pair of scissors to carefully dissect the surrounding tissues, taking care not to traumatize the vessel. The conical tip should always be directed above or below the vessel, never directly towards it.
  Prior ultrasound measurements and vein depth assessments should guide the harvester in approximating the vessel’s location. To avoid excessive tunnelling, it is essential to maintain the endoscopic scope in the same lateral plane as the patient. One helpful analogy is to envision a transverse CT scan of the leg, keep the tip of the scope aligned with that same transverse orientation. If muscle fibres become visible, it indicates that the dissection is too deep and should be corrected immediately.
3. Dissection of the vessel: Rapid and efficient dissection during EVH can be achieved through a structured, methodical approach typically involving anterior, lateral, and posterior dissection in sequence. However, in patients with thin legs or when harvesting from the lower leg, initiating dissection posteriorly in the tunnel is often advantageous. This technique creates a working cavity that allows the vein to suspend naturally, improving visualization and access for the bulky EVH device cannula shaft. Lateral dissection is then performed, followed by anterior dissection, which is generally more technically challenging. To minimize the risk of branch avulsion or microtears, the final stage may involve a controlled “sweeping” or “peel-off” technique, which gently separates the vein from the overlying superficial fascia. This stepwise approach enhances conduit preservation, particularly in anatomically delicate regions.^53^ Dissect surroundings as soon as possible and adipose tissues with the LSV and use the non-dominant hand to push for external manipulation for slippery or tough tissue dissection. Short and rotational, back and forth dissection techniques rather than forceful dissection will help to not traumatize the vessel wall. Distal branch dissection must be carried out using conical tips by creating windows on either side of the branches to be ligated. It is advised to ensure that the branches are at least 1cm in length to avoid any thermal spread during cauterization. The creation of lateral windows is suggested to be performed with the thin tip of the conical dissector, not the wider belly, as it could lead to tear the base of the vein branch.
4. Tip flip method: Use the lift port technique and flip the orientation from distal leg to proximal leg dissection. It is hard at the start and with experience you will be very proficient. Importantly never lose the site of the vein during tip flip and maintain the CO_2_ insufflation whenever possible.
5. Patient positioning for vein conduit: Elevating the thigh near the knee and allowing for external rotation of the leg, in combination with tilting the foot end down of the operating table, helps prevent the camera and cables from colliding with the ankle or abdomen. This positioning also facilitates smoother navigation of the saphenous vein, particularly around the knee or ankle region.
6. Blind spot: As a new EVH learner, it is important to understand the concept of blind spots which are the result of the EVH scope’s design (**Figure 1A and B**), where a portion of the instrument creates an invisible “shelf” along the scope. If unnoticed, this can cause the conduit to catch and increase the risk of endothelial injury, traction, or vessel perforation. Awareness of this blind spot and techniques to deal with it, like adjusting the scope position, can help reduce vein trauma and improve graft quality. To the authors’ knowledge, the Zimmer Biomet Venapax system is the only device specifically designed to eliminate all visual blind spots during endoscopic vein harvesting (**Figure 1C**).
7. Fasciotomy for thin legs: A fasciotomy (**Figure 2**) can be created to facilitate more space anteriorly or laterally for patients with thin legs. It is advised to always start the fasciotomy from the incision site and to move it forward distally. To assist further external compression can be delivered using the non-dominant hand to catch the fascia tissues on the Hemopro or bipolar ligating forceps. It is not always possible to go in a straight-line during fasciotomy so, rotation of the device can be used, if required, to create space wherever available inside the limb. The harvester should always take care near the branch site and continue in the direction of the next branch or do a tunnel turn to avoid tearing the base of the branch. Caution is imperative when operating near the underside of the dermis to prevent inadvertent thermal injury to the skin.
8. General trouble shooting:
  - Insufflation issues: Prior to initiating the procedure, it is essential to complete all basic safety checks to ensure optimal function of the equipment. These include verifying that the CO_2_ cylinder is adequately filled, the cylinder regulator is switched on, and the connection between the insufflator and the cylinder is secure. Additionally, the insufflator should be powered on and correctly set to the vein harvesting mode. These preparatory steps are critical for maintaining intraoperative safety and avoiding procedural delays or insufflation failure. In addition, check the line for kinks and disconnect it from the scope to ensure gas flow. If gas flows at the tubing/scope connection but the tunnel isn’t distended, the distal CO_2_ ports may be clogged. Flush them with saline if the device is in the patient, or with air if outside the patient. Check those points for any damage. If these steps do not work, we suggest disconnecting everything and reconnecting it.
  - Loss of tunnel or sucked tunnel: This is mainly caused by the fascia that has been divided around the muscle losing any seal and seeping the CO_2_ and causing the tunnel to collapse. Make sure for muscular patients whether it is upper or lower leg, do not cut the fascia on the posterior or lateral level. Sometimes, it is very easy to piece the fascia around the muscles with the conical tip, so take care while doing posterior dissection. If the space is confined and it is not viable to insert the EVH cannula shaft, it is suggested to do an anterior fasciotomy only. In addition, the harvester can try to stop the insufflation, so, remove all the equipment and squeeze the leg and re-introduce the equipment.
  - History of inguinal hernia: For patients with a history of inguinal hernia, avoid using that leg is possible. If not, stay at least 5 cm below the groin crease/inguinal ligament to avoid scrotal distension,^60^ in male patients.
  - Additional length for thicker branches: Expose large (>0.4 cm) branches as much as possible or in 2 areas one farther distally than you planned ligation site. If a stump continues to bleed after coagulation and cutting, it will limit the depth of the branch, causing it to retract into the fat. This will allow you to visualize the bleeding stump and re-cauterize it if necessary.

## COMMON PROBLEMS WITH ALL 3 SYSTEMS

1. In certain situations, fragile veins, particularly the superficial veins often found in women, can be easily damaged by a dissector due to their delicate nature. Dissecting these superficial veins can be particularly challenging, as the uneven surface of the harvester may obstruct its entry into the tunnel. Furthermore, smaller vein branches can become torn if resistance is encountered during dissection. Patient selection for EVH is vital during the learning curve period^9^^,^^32^^,^^52^^,^^5^,^8^
2. While dissecting a vein in the lower leg, the bulkiness of the device and the limited space for maneuverability can complicate access to the end of the tunnel, making it difficult to fully dissect the vein. Excessive twisting by the harvester can also disrupt smaller side branches. Reduced visibility can result from bleeding rather quickly due to the small tunnel space.
3. VasoView Hemopro 2, Terumo VirtuoSaph Plus, and Venapax Saphena Cannula sizes: The devices typically have a circumference ranging from 39 mm to 41 mm, which may be suboptimal for smaller patients and for use in the lower leg. Due to the bulkiness of the cannulas, there is a potential risk of exerting pressure, friction, and shear stress on the vessel wall and its branches. More exact measurements of each device are detailed in **Table 1**.
4. Any loss of visualization makes conduit harvesting nearly impossible. This may be due to bleeding within the tunnel, but another challenge is switching back to the dissector after harvesting without cleaning the endoscope.

**Table 1.** The Endoscopic Conduit Device Comparison Chart

Scope	Getinge Hemopro 2	Terumo Virtuosaph+	Zimmer Biomet Venapax
Length (working) of the dissector	42 cm	40.5 cm	39.5 cm
Dissector shaft width midpoint	7 mm	12.5 mm	12 mm
Conical tip width @ widest point	12.5 mm	13 mm	14.5 mm
Harvesting shaft cannula circumference	40 mm	41 mm	39 mm
Conical tip angle	30°	33°	41°
Flare distance	33 mm	22 mm	19 mm

## WHAT SHOULD AN IDEAL EVH SYSTEM LOOK LIKE?

We propose that the ideal EVH system should function as an all-in-one platform, integrating key features to streamline the procedure while reducing the learning curve. It should support standardized training across various devices, minimize thermal spread, incorporate advanced dissection tools, and apply “non-touch” technology to optimize vessel preservation.

A short learning curve is crucial for EVH adoption. Contributing design elements include:

1. *Simplified setup*: few components and straightforward assembly reduce the cognitive and physical burden.
2. *Ergonomic design*: intuitive hand controls reduce strain on the wrist, fingers, and shoulder, improving manipulation and precision.
3. *Simulation compatibility*: integration with virtual or enhanced simulation platforms facilitates skill acquisition without immediate patient exposure.
4. *Integrated visual feedback*: high-definition, real-time imaging enhances anatomical recognition and dissection safety.
  - Dissection tool should be slim, multifunctional, and capable of atraumatic dissection, coagulation, retraction, and mobilization. Haptic feedback can enhance operator control and safety. Reducing instrument exchanges may further improve efficiency and learning outcomes.
  - Non-touch technology is essential for preserving vessel integrity. This involves avoiding direct manipulation with hooks or stabilizers and preserving a thin layer of perivascular fat to protect the adventitia. Advanced systems should also seal side branches without contacting the vein, limiting thermal and mechanical trauma, particularly during early operator experience.^53^^,^^57^
  - Additional essential features include smoke and fluid evacuation, and modularity to accommodate different patient sizes (eg, variable limb circumference, BMI). Tailored cannula lengths and diameters are especially important for thin limbs or lower leg and RA harvesting, where smaller anatomical spaces increase technical complexity. While assumptions of ethnic tissue differences are scientifically challenged, interindividual anatomical variability remains clinically significant.
  - Data integration for tracking the learning curve is also critical. Systems should record parameters such as harvesting time, CO_2_ insufflation pressure, diathermy settings, and user characteristics. Vein quality scoring tools (eg, assessing bruising, avulsions, and graft usability) should be embedded to evaluate procedural outcomes.
  - Integration of real-time video feedback enhanced by AI offers potential for objective analysis of dissection technique, equipment handling, applied pressure, and complication identification (eg, bleeding or branch tears). AI-assisted feedback may help identify performance gaps and accelerate technical proficiency, particularly valuable where one-to-one mentoring is limited. Although initial costs may rise, such capabilities provide substantial value during the early learning period.
  - Ideal EVH system should be supported by a comprehensive, standardized training programme including simulation-based early-stage training, structured mentorship, and a train-the-trainer component. Novice operators should train in dry and wet lab environments prior to patient contact to ensure safety and competence.

Finally, device design must evolve towards sustainability. Manufacturers should prioritize hybrid, reusable, or recyclable systems to reduce both environmental impact and long-term disposable costs.

## DISCUSSION

A meta-analysis by the International Society for Minimally Invasive Cardiothoracic Surgery (ISMICS), encompassing 76 controlled randomized and non-randomized studies published between 2005 and 2015 which concluded that endoscopically harvested saphenous veins and radial arteries were of non-inferior quality to the open vessel technique conduits.^32^ There are many variables which can cause post-surgery conduit failure and bypass graft occlusion. These include the normal pathological changes (intimal proliferation, smooth muscle migration, dilatation of the conduit) once it has been grafted into the arterial system^62^ and the surgical technique used to harvest the conduit. The patency rate of LSV during the first-year ranges from 75% to 86% and decreases to 55% to 60% in the long term.^61^^,^^62^

As a collective of experts in harvesting, we believe that graft failure can be affected by harvesting techniques including trauma to the conduit during the EVH learning curve and harvester’s training level and experience. This trauma may act independently or as an accelerating factor alongside other well-established risk factors contributing to pathological changes in the conduit following its use in CABG surgery. These changes include intimal hyperplasia, smooth muscle cell migration, and conduit dilatation.

We recommend that further research in this area is essential and that surgical device manufacturers, healthcare providers, and educational institutions should invest in internationally standardized training programmes. These programmes should adopt a co-production methodology involving clinicians, patients, device manufacturers, and educators and should incorporate ongoing mentorship. Such an approach is crucial to minimizing the impact of the learning curve on patient outcomes in CABG surgery.

We suggest that it is very important for trainee harvesters to develop the ability to assess the quality of the vein using ultrasound, as this enables them to make informed decisions, in collaboration with the multidisciplinary team, about which conduit to harvest. The use of a vein scoring tool is particularly important, as it allows for the tracking of their conduit harvesting patterns and provides an objective measure of conduit quality, ensuring that the best possible graft is selected for the patient. The scoring tool not only enhances the accuracy of conduit selection but also supports continuous improvement in the trainees’ skills and decision-making. An example of a vein scoring tool developed by a multidisciplinary team is provided in **Table S3**.

## ENVIRONMENTAL IMPACT OF DISPOSABLE vs REUSABLE OR HYBRID EVH DEVICES

Anthropogenic greenhouse gas (GHG) emissions, to which healthcare contributes 4%-8% in high-income countries, exacerbate global warming and associated health risks, including respiratory and infectious diseases.^63^ Although EVH offers clinical advantages with reduced postoperative pain, better cosmetic outcomes, and higher patient satisfaction,^23^^,^^57^^,^^58^ its environmental footprint remains underexplored. Single-use instruments, commonly employed in EVH, are significant sources of solid waste and GHGs compared to reusable systems^63–65^; however, reusables also incur environmental costs due to energy- and water-intensive sterilization.^66^ For instance, reusable laryngoscopes may emit 3 times more CO_2_ and consume 10-fold more water than single use equivalents.^67^

EVH devices, with complex components, present cleaning challenges that may elevate infection risks. Anecdotal reports from Europe and Asia suggest some hospitals chemically sterilize and reuse single-use EVH devices up to 20 times, but no peer-reviewed studies validate the safety or performance of this practice. Reusability depends on safe reuse cycles, sterilization efficiency, energy source, and regulatory compliance with FDA or MHRA standards. Hybrid systems may offer a viable compromise: during cholecystectomy, hybrid laparoscopic instruments reduced carbon emissions significantly (1.756 vs 7.194 kgCO_2_e), and hybrid robotic ports achieved 83% lower emissions compared to disposable counterparts.^63–68^ However, data specific to EVH are lacking. Only 2 studies have quantified cardiovascular surgery emissions (124-505 kgCO_2_e per case),^64^ and EVH likely adds further burden due to disposable kits and CO_2_ insufflation (∼0.9 kgCO_2_e per cylinder). Future research must prioritize EVH-specific LCAs and outcome-linked sustainability analyses to guide evidence-based, environmentally responsible surgical practices.^63–69^

## CONCLUSION

This review provides an overview of the current evidence on the advantages, limitations, and clinical relevance of various EVH systems. The existing literature does not support the superiority of one EVH device or technique over another. Notably, harvester preference appears to be influenced more by institutional exposure and training background than by comparative outcome data or cost-effectiveness.

All currently available EVH systems present unique strengths and limitations. We recommend that practitioners attain proficiency in one system before transitioning to another to minimize technical errors and optimize patient outcomes. Selection of an EVH platform should be based on clinical performance, safety profile, long-term graft patency, and institutional needs, rather than historical use alone.

To support evidence-based decision-making and improve patient outcomes, we advocate for the establishment of an international EVH registry or integration of EVH-specific data such as device type and harvester experience into existing national cardiac surgery databases.

## Supplementary Material

ivaf204_Supplementary_Data

## Data Availability

Due to nature of this review, no data were used for the research described in the article.
