# Supplementary material for: An Expert Panel Review of Endoscopic Vein Harvesting Devices: Benefits, Limitations, and Clinical Insights
Source: Interdiscip Cardiovasc Thorac Surg. 2025 Sep 2;40(9):ivaf204. doi: 10.1093/icvts/ivaf204 (PMC12548038; doi:10.1093/icvts/ivaf204)
Supplement: ivaf204_Supplementary_Data [file ivaf204_Supplementary_Data.zip › Suppl Table 1 Illustrates different endoscopic systems.docx]

**Supplementary Table 1: Illustrates different endoscopic systems and its evolution**

| **System** | **Name of the device** | **Year introduced** | **Rationale for its evolution** |
| --- | --- | --- | --- |
| **Getinge/Maquet**   - Single use - Closed or Open tunnel CO2 system | VasoView 1 | 1996 | - First Endoscopic vein harvesting system and first procedure performed by Dr. Al Chin in Belgium. - Bipolar cautery and Zero-degree endoscope. |
|  | VasoView 2 | 1997 | - Improved visualisation and ergonomics |
|  | VasoView 3 | 1998 | - Enhanced video technology and refined instruments. First in-line EVH system developed. |
|  | VasoView 4 | 1999 | - More streamlined technology product. |
|  | VasoView 5 | 2002 | - Integrated system |
|  | VasoView 6 | 2004 | - Continued refinement with High Definition (HD) imaging and ergonomic enhancements - Allows proximal and distal CO_2_ insufflation - Bisector bipolar ligating forceps tips for cauterisation and ligation simultaneously - Hand controlled buttons to eliminate wrapping or tangling |
|  | VasoView 7 | 2006 | - VV7 scissors ligating forceps tips for cauterisation and ligation simultaneously. |
|  | HaemoPro 1 and 1.5 | 2006  Withdrawn Sep 2024 | - Advanced camera technology with better vein preservation - Harmonic scalpel technology to reduce the thermal spread to the vein - Withdrawn due to risk that silicone may detach from the harvesting tool (17 reported cases worldwide), no reports of death or serious injuries. |
|  | HaemoPro 2 | 2011 to present | - Virtually minimising thermal spread to help harvesters safely acquire high-quality conduits. - Cut and seal technology with superb visualisation and manoeuvrability. - Issues reported C-ring and harvesting tool is closer than normal (27 complaints raised worldwide). |
|  | HaemoPro 3 | 2024 to present | - Enhanced smoke evacuation, regulated energy control, ergonomic game controller style handle with an integrated cable. |
| **Terumo Corporation**   - Single use   Open tunnel CO2 system | Terumo EVH system 1 | 1999 to 2001 | - To reduce wound complications and improve better patient satisfaction |
|  | Terumo EVH system 2 | 2008 to 2015 | - New key features to increase its precision and improve ergonomics |
|  | Terumo Virtuo Saph Plus | 2015 to present | - Terumo’s V-keeper with a V-lock and V-cut system to stabilise the vein and adjust the distance between the cautery-cut device and vessel wall. - V-cutter safeguards the main blood vessel from thermal damage while side branches are getting ligated. - V-Keeper with lock system designed to hold the veins securely and enhance the manipulation and visualisation of side branches. |
| **Karl Storz**   - Reusable   Open tunnel CO2 system | Karl Storz Endoscopic Vessel Harvester | 2002 to present | - Less pressure on the vessel due to the open tunnel CO2 harvesting system. - More cost-effective due to the reusable equipment. - Two-hand technology puts pressure on both harvester’s shoulder. - Steep learning curve and the risk of muscular injury due to the strength required to lift and manoeuvre the equipment during harvesting. |
| **Zimmer Medical**   - Single use - Closed CO2 system | Venapax EVH device | 2013 to present | - There is no C-ring, which significantly reduces contact with the vein and potential damage to it. - The novel unitary technology with retractable ultra-thin bipolar blades allows harvesters to create the tunnel, dissect, and ligate branches simultaneously. - The spot cauterisation technique during dissection helps reduce bleeding. - The 2-pass technique reduces contact with the vein and allows for a single entry into the leg. |
