# Supplementary material for: An Expert Panel Review of Endoscopic Vein Harvesting Devices: Benefits, Limitations, and Clinical Insights
Source: Interdiscip Cardiovasc Thorac Surg. 2025 Sep 2;40(9):ivaf204. doi: 10.1093/icvts/ivaf204 (PMC12548038; doi:10.1093/icvts/ivaf204)
Supplement: ivaf204_Supplementary_Data [file ivaf204_Supplementary_Data.zip › Suppl Table 2- illustrates advantages, disadvantages .docx]

**Supplementary Table 2: Illustrates the advantages, disadvantages and expert tips of EVH devices**

| **EVH system** | **Type of instrument** | **Advantages** | **Disadvantages** | **Expert tips** |
| --- | --- | --- | --- | --- |
| **Dissection Tip (Tunnelling or vein dissection tool)** | Getinge | - The blunt, crystal-clear cone tip facilitates tissue dissection and enhances visualisation. - A larger proximal bulb allows for effective dilation of the subcutaneous tunnel. | - Excessive shearing force during dissection may result in intimal injury, including tears or abrasions. - In challenging dissections, inadvertent vein puncture may occur, increasing the risk of CO₂ embolism. - Improper tightening onto the endoscope can compromise device integrity. - In patients with minimal subcutaneous tissue, especially those with thin legs, the limited space between the proximal bulb and vein may increase the risk of adventitial damage. | - Maintain a tilt of the cone tip away from the vein to reduce the risk of vessel injury. - Avoid forceful advancement; instead, use external manipulation with the non-dominant hand to guide tissues toward the dissector. - Employ short, rotational movements in both forward and reverse directions for controlled dissection. - Create sufficient fascial windows (≥1 cm) on either side of vein branches using external manipulation to optimise branch exposure and develop “Y” configurations. |
|  | Terumo | - The tapered distal tip (12.5 mm diameter) facilitates atraumatic separation of surrounding tissue from the target vessel and its branches. - The PTFE sheath minimises friction and allows smooth mobility around the endoscope. - An open CO₂ insufflation system delivers gas directly to the working space, supporting tunnel maintenance and visibility. | - The device may inadvertently damage superficial side branches during dissection. - Its bulkiness reduces manoeuvrability, particularly in the lower leg where subcutaneous space is limited. - Reintroducing the dissector after electrocautery may result in fluid ingress, compromising visual clarity. - Distal insufflation ports can become occluded by adipose tissue or blood, disrupting CO₂ flow and causing tunnel collapse. | - Trocar use is optional and can be adjusted based on operator preference. - Wrapping the PTFE sheath with a laparotomy sponge may enhance grip during manipulation. - External pressure applied to the tunnel can minimise longitudinal torque on the dissector. - If insufflation ports become blocked, flushing the CO₂ connector with saline may help clear obstructions and restore function. |
|  | Zimmer Venapax | - Features a blunt, transparent conical tip to facilitate safe dissection. - A frosted marking on the tip provides a visual guide for the emergence point of the retractable blades, aiding in precise spot cautery and branch ligation. - Enables simultaneous dissection, branch cauterisation, and ligation, potentially improving procedural efficiency. | - The retractable blade window, positioned behind the conical tip, may accumulate blood, which can obscure visibility if it leaks into the field. - The integrated design may create a sensation of increased resistance during initial use due to the internal mechanics housed within the shaft; however, this typically resolves as users adapt to the device's handling characteristics. | - To maintain visibility, periodically rotate the tip in clean adipose tissue or dissect through fresh tissue to clear blood or condensation. - Avoid the use of anti-fog solutions on the conical tip, as it is plastic rather than glass and may become clouded. - Awareness of the blade emergence zone is critical to avoid inadvertent vessel injury during activation. |
| **C-ring (Vein retraction tool)** | Getinge | - The integrated C-ring assists in stabilising the pedicle and maintaining an optimal 3–4 mm distance from the vessel during cauterisation. - While the manufacturer recommends minimising C-ring use, its stability may benefit experienced users under specific conditions; however, it may be less suitable for novice practitioners. | - Manipulation of the vein and its branches using the C-ring can generate shear stress, particularly when performed by less experienced harvesters. - Effective operation requires substantial experience to manage full system rotation without applying excessive traction to the vein, increasing the risk of vessel trauma. | - Minimise use of the C-ring where possible to reduce the risk of mechanical stress on the vessel. - Avoid twisting or pulling on vessel branches with the C-ring. If better exposure is needed, remove and reposition the C-ring rather than forcing rotation or twisting. - Emphasise precise handling and use of adjunctive external tissue manipulation to maintain branch visibility and vessel integrity. |
|  | Terumo (vein keeper and lock) | - Locking technology to protect the vein. | - The locking mechanism may inadvertently apply rotational torque or traction on the conduit if not carefully managed, particularly by less experienced operators. - Proficient handling is required to coordinate system-wide movement, rotating the V-keeper, advancing the cautery device, and simultaneously protecting the conduit. - Sometimes, the V-keeper does not fully retract, possibly limiting visibility of side branches and remaining connective tissue, which may increase the risk of bleeding within the tunnel. - The V-keeper mechanism does not completely retract which may hinder scope insertion among novice harvesters. | - Employ a forward-dissection technique to maintain consistent tunnel progression. - Use the V-keeper’s structure to assist with lifting tunnel tissue during scope advancement. - Ensure tension is directed onto the side branches, not the main conduit, during harvesting. - During cauterisation, apply the "engage, ground, cut" technique: - **Engage:** Position the branch within the V-cutter. - **Ground:** Stabilise the grounding pads on the outside of the V-cutter against the tunnel wall. - **Cut:** Activate cautery with gentle forward pressure until the branch detaches cleanly. |
|  | Zimmer Venapax | - No C-ring by design, to reduce contact with the vein. - Eliminates the need for grabbing, twisting, and torquing the vein at the base of the branches. | - A harvester with experience using a legacy device must learn to harvest the vein without the C-ring. This is not an issue for a new harvester. | - Perform posterior dissection first, taking branches as you go along. This will suspend the vein inside the tunnel, making anterior dissection easier. |
| **Hemopro technology (Branch cutting/coagulation)** | Getinge | - The concave inner surface of the Hemopro jaws offers a broad insulated area, enhancing safety during cauterisation. - A built-in safety shut-off mechanism deactivates energy delivery after prolonged activation (typically beyond 14 seconds), reducing the risk of thermal injury. - Manufacturer guidelines recommend power setting 3 for effective vessel sealing and transection. | - The concave design of the jaws can complicate the division of superficial or variably sized branches, particularly near the skin surface, and may increase the risk of thermal injury to the vein wall especially when dividing complex "Y" branches. - Following the safety shut-off, a 30-second cooling period is required before reactivation, potentially delaying branch division during thick tissue dissection. - Power setting 3 may cause carbonisation or blackening of the vein adventitia. Adjustments in power setting (between 2 and 3) are advised based on patient anatomy and vessel characteristics to minimise thermal damage. | - Avoid cauterising surrounding fat, as this generates smoke that can obscure visibility. In patients with larger limbs, tapping the skin or briefly pausing (~10 seconds) can aid smoke dissipation. Alternatively, a 2-inch needle may be inserted into the tunnel to allow smoke evacuation. - Blind cauterisation should be avoided to prevent inadvertent injury to the vessel wall or complete transection of the vein. - Prolonged activation in contact with surrounding tissue may lead to overheating, potentially melting the silicone insulation sheet and causing detachment of the cauterising element, rendering the device non-functional. |
| **V-cutter (Bipolar cautery)** | Terumo (V-cutter) | - Designed to protect the conduit from thermal injury during side branch division by enabling cautery to be grounded into the tunnel wall, minimising thermal spread. - Allows for blunt tissue dissection without activation of diathermy. - The spacing between V-cutter blades promotes optimal tissue compression, facilitating haemostatic sealing. - Reversible current functionality allows the grounding plates on the outside of the V-cutter to serve as spot cautery when needed. | - Larger branches occasionally pose a problem in the V-cutter due to the location of the cautery element. Care must be taken to allow for careful sealing of large vessels. - Due to the grounding design diverting energy away from the conduit, large branches may bleed from the conduit side after division, possibly obscuring view. | - Use short bursts of cautery and gentle forward pressure for effective sealing; larger branches may require a stab incision or ligature. - Exercise caution when using spot cautery near deep dermal layers to avoid unintended tissue injury. - Avoid pulling or dragging the conduit through the V-keeper; instead, use the device to stabilise the vein and align branches appropriately with the cutter. - The V-keeper should be reserved for branch division and is not necessary for cutting connective tissues. - Larger branches are best addressed at the end of the procedure to reduce the risk of prolonged bleeding. - In the event of significant bleeding, withdraw the system and apply external compression to control haemorrhage. |
| **Bipolar cautery** | Zimmer Venapax | - Thin and sleek bipolar electrodes for sealing and ligating branches. - Thermal spread is less than 1mm. - The blades can also be used to spot cauterise and divide fascia. | - There is a learning curve to master the use of bipolar cautery for sealing and taking branches. - It requires learning the correct angles and how to apply slight tension to the branch distally while ligating it. | - Minimal dissection is required, with only the tip of the cone around the distal branch, creating a tiny window to slide the blades of the bipolar inside to seal and cut the branches. - A slight rotation (1-2mm) of the branch, when between the blades, creates tension to assist in dividing the branch. - A slight advancement of the blades after sealing the branch helps to divide it. |
| **Additional features** | Terumo | - The wiper on the camera lens cleans the camera and improves visibility. - The open CO_2_ system allows for "stab and grab" to be performed earlier. - Circumferential control of the vessel with the V-keeper prevents the need to "re-run" the vein to check for missed tissue. - The spot cautery function helps control bleeding on the tunnel wall. | | |
|  | Zimmer Venapax | - The unitary dual-pass system eradicates double entry, especially on large obese legs. | | |
