# Supplementary material for: An Expert Panel Review of Endoscopic Vein Harvesting Devices: Benefits, Limitations, and Clinical Insights
Source: Interdiscip Cardiovasc Thorac Surg. 2025 Sep 2;40(9):ivaf204. doi: 10.1093/icvts/ivaf204 (PMC12548038; doi:10.1093/icvts/ivaf204)
Supplement: ivaf204_Supplementary_Data [file ivaf204_Supplementary_Data.zip › Suppl table 3 Vein scoring system .docx]

**Supplemental table 3: Vein scoring system for vein harvesting techniques**

| **Quality of the vein** | **Type 1:** Small calibre  (<2mm to 3mm) | | | **Type 2:** Normal calibre  (3mm to 5mm) | | | **Type 3:** Large calibre  (5mm to >8mm) | | |
| --- | --- | --- | --- | --- | --- | --- | --- | --- | --- |
| **Size** |  |  | Score |  |  | Score |  |  | Score |
|  | 2mm - 3mm | 2 |  | 3mm - 4mm | 0 |  | 5mm - 8mm | 4 |  |
|  | < 2mm | 3 |  | 4mm - 5mm | 1 |  | > 8mm | 5 |  |
| **Bruising/haematoma** | None | 0 |  | None | 0 |  | None | 0 |  |
|  | Mild (<25%) | 1 |  | Mild (<25%) | 1 |  | Mild (<25%) | 1 |  |
|  | Severe (>25%) | 2 |  | Severe (>25%) | 2 |  | Severe (>25%) | 2 |  |
| **Repair**  **(avulsions)** | None | 0 |  | None | 0 |  | None | 0 |  |
|  | 1 to 2 | 1 |  | 1 to 2 | 1 |  | 1 to 2 | 1 |  |
|  | 3 to 4 | 2 |  | 3 to 4 | 2 |  | 3 to 4 | 2 |  |
|  | >4 | 3 |  | >4 | 3 |  | >4 | 3 |  |
|  | Unable to repair | 4 |  | Unable to repair | 4 |  | Unable to repair | 4 |  |
| **Branch cut short /**  **Tie too close to the vein** | 3 - 5mm | 0 |  | 3 - 5mm | 0 |  | 3 - 5mm | 0 |  |
|  | < 3mm | 1 |  | < 3mm | 1 |  | < 3mm | 1 |  |
|  | <1mm | 2 |  | <1mm | 2 |  | <1mm | 2 |  |
| **Varicose veins** | None | 0 |  | None | 0 |  | None | 0 |  |
|  | Mild (3 – 5 entire length) | 1 |  | Mild (3 – 5 entire length) | 1 |  | Mild (3 – 5 entire length) | 1 |  |
|  | Multiple (> 6) | 2 |  | Multiple (> 6) | 2 |  | Multiple (> 6) | 2 |  |
| **Total score** |  | |  |  | |  |  | |  |

- The highest score may be the grounds for rejection of vein graft but if there is no other conduit availability, the highest score veins can be used under Consultant Cardiac surgeon’s discretion.
- Type of veins used on the coronaries: OM – Type 1 PDA -Type 1 Diagonal - Type 1 LAD – Type 1 RCA – Type 1

Type 2 Type 2 Type 2 Type 2 Type 2

Type 3 Type 3 Type 3 Type 3 Type 3

LIMA/RIMA Radial artery
