# Supplementary figures and images for: An Expert Panel Review of Endoscopic Vein Harvesting Devices: Benefits, Limitations, and Clinical Insights
Source: Interdiscip Cardiovasc Thorac Surg. 2025 Sep 2;40(9):ivaf204. doi: 10.1093/icvts/ivaf204 (PMC12548038; doi:10.1093/icvts/ivaf204)

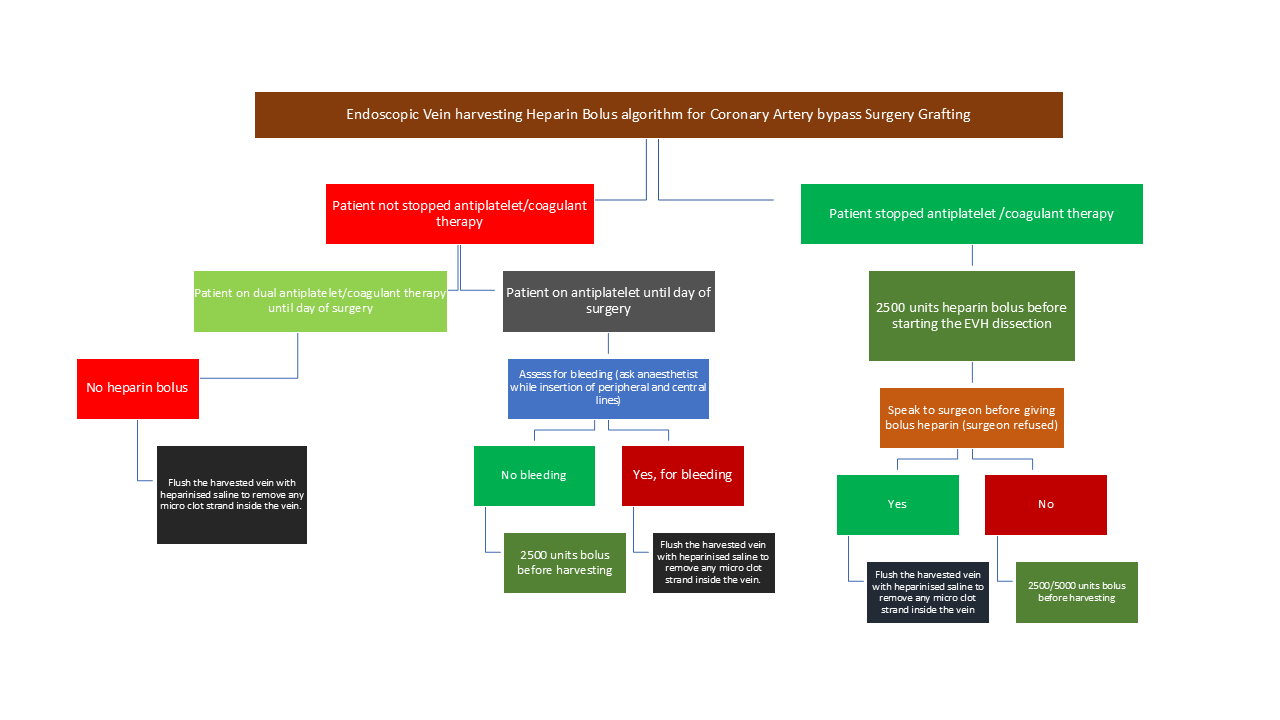

Supplement: ivaf204_Supplementary_Data [file ivaf204_Supplementary_Data.zip › Suppl Fig 1 EVH Heparin Bolus algorithm.tif]
